# Causal analysis approaches in Ingenuity Pathway Analysis

**DOI:** 10.1093/bioinformatics/btt703

**Published:** 2013-12-13

**Authors:** Andreas Krämer, Jeff Green, Jack Pollard, Stuart Tugendreich

**Affiliations:** ^1^Ingenuity Systems, 1700 Seaport Boulevard, Redwood City, CA and ^2^Translational and Experimental Medicine—Bioinformatics, Sanofi-Aventis, 270 Albany Street, Cambridge, MA, USA

## Abstract

**Motivation:** Prior biological knowledge greatly facilitates the meaningful interpretation of gene-expression data. Causal networks constructed from individual relationships curated from the literature are particularly suited for this task, since they create mechanistic hypotheses that explain the expression changes observed in datasets.

**Results:** We present and discuss a suite of algorithms and tools for inferring and scoring regulator networks upstream of gene-expression data based on a large-scale causal network derived from the Ingenuity Knowledge Base. We extend the method to predict downstream effects on biological functions and diseases and demonstrate the validity of our approach by applying it to example datasets.

**Availability:** The causal analytics tools ‘Upstream Regulator Analysis', ‘Mechanistic Networks', ‘Causal Network Analysis' and ‘Downstream Effects Analysis' are implemented and available within Ingenuity Pathway Analysis (IPA, http://www.ingenuity.com).

**Supplementary information:** Supplementary material is available at *Bioinformatics* online.

## 1 INTRODUCTION

The interpretation of high-throughput gene-expression data is greatly facilitated by the consideration of prior biological knowledge. Traditionally this has been done using statistical gene-set-enrichment methods where differentially expressed genes are intersected with sets of genes that are associated with a particular biological function or pathway (Abatangelo *et al.*, 2009). One more recent approach involves the application of causal networks that integrate previously observed cause–effect relationships reported in the literature (Chindelevitch *et al.*, 2012a; Felciano *et al.*, 2013; Kumar *et al.* 2010; Martin *et al.*, 2012; Pollard *et al.*, 2005). While still depending on statistics, this is more powerful than gene-set enrichment since it leverages knowledge about the direction of effects rather than mere associations. In this article, we describe causal analysis approaches that have been implemented in Ingenuity Pathway Analysis (IPA) with particular focus on the details of the underlying algorithms, and the application to a number of real-world use cases. IPA is a commercial software package and is described in the supplementary material.

Given a gene-expression dataset, our main goals are to elucidate the upstream biological causes and probable downstream effects on cellular and organismal biology. It is critical to infer the identity of upstream regulatory molecules and associated mechanisms to provide biological insight to the observed expression changes. We also aim to predict whether such regulators are activated or inhibited based on the distinct up- and down-regulation pattern of the expressed genes, and determine which causal relationships previously reported in the literature are likely relevant for the biological mechanisms underlying the data. Upstream regulators are not limited to transcription factors; they can be any gene or small molecule that has been observed experimentally to affect gene expression in some direct or indirect way. A similar approach, relying on the same methodology is also used to predict downstream functional effects and phenotypes. Apart from generating likely mechanistic hypotheses, causal inference can also be used to find potential upstream regulators with a response opposite to the observed expression pattern, which is useful for the prediction of therapeutic compound effects. This application is similar to the approach taken by the Connectivity Map tool (Lamb *et al.*, 2006), except that here we rely on the wide range of literature-curated biological findings about compounds and their interactions instead of a gene-expression database derived from *in vitro* tested compounds.

The causal network underlying our algorithms is based on the Ingenuity Knowledge Base, a large structured collection of observations in various experimental contexts with nearly 5 million findings manually curated from the biomedical literature or integrated from third-party databases. The network contains ∼40 000 nodes that represent mammalian genes and their products, chemical compounds, microRNA molecules and biological functions. Nodes are connected by ∼1 480 000 edges representing experimentally observed cause–effect relationships that relate to expression, transcription, activation, molecular modification and transport as well as binding events. Network edges are also associated with a direction of the causal effect, i.e. either activating or inhibiting.

We describe four causal analytics algorithms that are available in IPA: (i) Upstream Regulator Analysis (URA) determines likely upstream regulators that are connected to dataset genes through a set of direct or indirect relationships; (ii) Mechanistic Networks (MN) builds on URA by connecting regulators that are likely part of the same signaling or causal mechanism in hypothesis networks; (iii) Causal Network Analysis (CNA) is a generalization of URA that connects upstream regulators to dataset molecules but takes advantage of paths that involve more than one link (i.e. through intermediate regulators), and can be used to generate a more complete picture of possible root causes for the observed expression changes; and (iv) Downstream Effects Analysis (DEA) applies the methodology of URA to the inference of and impact on biological functions and diseases that are downstream of the genes whose expression has been altered in a dataset. Using several examples we show how these tools are applied to gene-expression data in practice.

## 2 APPROACH

The inference of upstream regulators needs to be based on statistics since it cannot be guaranteed that all relationships present in the causal network are relevant and actually occur in the given experimental context. Also, genes are often modulated by several upstream regulators (sometimes with opposing effects), and it is not known *a priori* which will dominate in a particular system. Filtering literature findings by specific contexts (e.g. by a particular tissue or cell line) generally does not work well because it leads to networks that are too sparse for meaningful inference. We therefore construct many possible upstream regulators and networks serving as hypotheses for the actual biological mechanism underlying the data, and then score those regulators by their statistical significance. In particular, we use two scores that address two independent aspects of the inference problem: an ‘enrichment’ score [Fisher’s exact test (FET) *P*-value] that measures overlap of observed and predicted regulated gene sets, and a *Z*-score assessing the match of observed and predicted up/down regulation patterns. We find that a *Z*-score is particularly suited for this kind of problem since it serves as both a significance measure and a predictor for the activation state of the regulator.

A similar approach has been reported in Pollard *et al.* (2005) where ‘richness’ and ‘concordance’ *P*-values are used to score regulators of expression changes derived from type 2 diabetes patients. More recently Chindelevitch *et al.* (2012a,b) present a rigorous discussion of statistical significance in a causal network with signed interactions based on a ‘ternary dot product distribution’. This is achieved by transforming the network such that edge signs are projected onto the nodes, and exact *P*-values are computed separately for both possible activation states of a given upstream regulator. In contrast, the *Z*-score used in the present approach, represents an (asymptotic Gaussian) approximation, but is easier to compute and combines both cases into one score.

## 3 METHODS

The various causal analysis methods used in IPA are described below.

### 3.1 Causal network

Causal analysis algorithms are based on a ‘master’ network which is derived from the Ingenuity Knowledge Base, and given by a directed multigraph 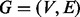 with nodes 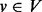 representing mammalian genes, chemicals, protein families, complexes, microRNA species and biological processes, and edges 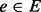 reflecting observed cause–effect relationships. For the following let 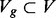 be the set of all genes, and 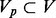 the set of all biological processes. For each edge 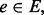 we define functions 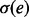 and 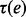 that map 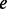 to its unique source and target nodes, respectively. The graph 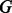 has no self-edges, i.e. 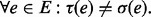 Each edge in 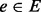 is associated with a set of underlying findings 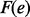 obtained from the literature, where each finding 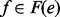 is associated with a ‘sign’ 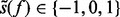 that represents the regulation direction of the causal effect. If 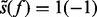 effect is activating (inhibiting), and for 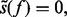 the direction of the effect is unknown or ambiguous. Depending on the underlying findings, edges are classified into the distinct types, ‘T’, ‘A’ and ‘P’, represented by three disjoint subsets of *E*: *E_t_*, *E_a_* and *E_p_*. T-edges are related to transcription and expression events including protein–DNA binding (i.e. regulation of the abundance of the target node), while A-edges represent the functional activation or inhibition of the target node (e.g. through phosphorylation in a signaling cascade). P-edges are associated with the regulation of biological processes (e.g. apoptosis). The master network *G* is a multigraph since two given source and target nodes can be connected by a T-edge, and an A-edge at the same time.

The various finding categories and their respective association with edge types and signs are given in a table in the Supplementary Material. Findings about changes of molecular modification states (e.g. phosphorylation) are included in the A-edge type if an activating or inhibiting effect can be inferred. All T-edges are connected to genes as their target nodes, 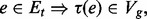 and all P-edges connect to biological processes, 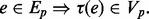 Depending on the signs of the underlying findings, each edge 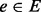 is in turn associated with a unique direction of the causal effect that is either activating, inhibiting or unknown, and represented by the sign 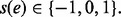 In addition, we also associate edges with weights 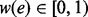 reflecting our confidence in the assigned direction of the effect. Details are given in the Supplementary Material.

### 3.2 Gene-expression data

All differentially expressed genes in a given dataset that are also present as nodes in the master network form a subset 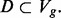 The methods described here do not take individual expression levels into account but instead assume that transcriptionally altered genes have been determined using a suitable cut off applied to the measured expression change. Each gene in the dataset, 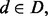 can be either up- or down-regulated which is represented by the sign 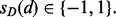


### 3.3 Upstream Regulator Analysis

The goal of URA is to identify molecules upstream of the genes in the dataset that potentially explain the observed expression changes. Since it is *a priori* unknown which causal edges in the master network are applicable to the experimental context, we use a statistical approach to determine and score those regulators whose network connections to dataset genes as well as associated regulation directions are unlikely to occur in a random model. In particular, we define an overlap *P*-value measuring enrichment of network-regulated genes in the dataset, as well as an activation *Z*-score which can be used to find likely regulating molecules based on a statistically significant pattern match of up- and down-regulation, and also to predict the activation state (either activated or inhibited) of a putative regulator.

Here, we consider transcription and expression (T) edges only by looking at the subgraph 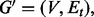 and defining the subset of genes that are regulated by at least one edge in 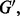


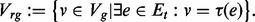

A potential regulator *r* can be any node in *V* that is either a gene, protein family, complex, microRNA, or chemical. For a particular given regulator 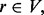 we define the set of downstream regulated genes as

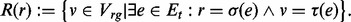

For each 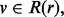 the sign of *v* is defined as regulation direction of *v* under the assumption that *r* is activated, which is given by the regulation direction of the connecting edge, as

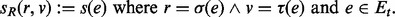

Similarly we define the weight associated with *v* to be

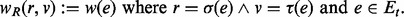



#### 3.3.1 Overlap P-value

**Fig. 1.**
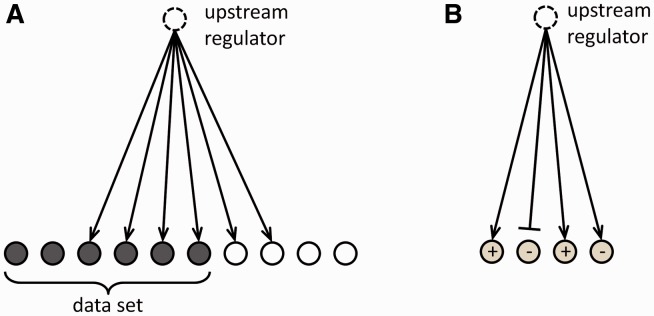
Overlap *P*-value (A) and activation *Z*-score (B) calculation (see text). In (B) the pointed arrowheads represent activating relationships, and the blunt arrowheads represent inhibitory relationships

For a particular regulator *r* the overlap *P*-value *p*(*r*) measures enrichment of *r*-regulated genes in the dataset *D* without taking into account the regulation direction, i.e. independent of the edge weight or sign (Fig. 1A). Protein–DNA-binding edges with sign equal to zero are included. The calculation is based on the one-sided FET which assumes a random dataset with a constant number of genes as the null model, and where the *P*-value is given by

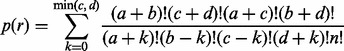

where 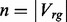 is the size of the ‘universe’ *V_rg_*, the ‘overlap’ is given by 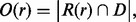 and 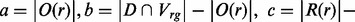 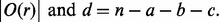

#### 3.3.2. Activation Z*-score*

The activation *Z*-score makes predictions about potential regulators by using information about the direction of gene regulation. Its purpose is two-fold: first, it can be used to infer the activation state of a putative regulator (i.e. whether the regulator is activated or inhibited). This is achieved by assessing consistency of the pattern match between the up/down gene-regulation pattern and the activation/inhibition pattern given by the network relative to a random pattern. Second, similar to the overlap *P*-value, the *Z*-score can be used to determine likely regulators based on statistical significance of the pattern match. However, the latter use requires careful assessment whether the underlying null model is appropriate, which is discussed in more detail below. For the purpose of the activation *Z*-score we only consider edges where the regulation direction is well-defined, i.e. 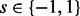 (Fig. 1B). For a particular regulator *r* the ‘overlap’ between *r*-regulated genes and the dataset is then given by

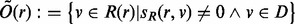

and we define the activation *Z*-score as

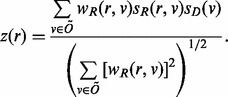

As shown below *z*(*r*) is approximately normally distributed for random signs 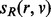 or 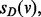 so we can interpret *z* in the following way: if 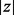 is high enough (say 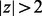) we consider the match between the signs 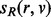 and 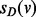 as being significant. The sign of *z* then determines whether the regulator *r* is predicted to be activated or inhibited. For 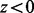 the signs 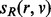 and 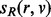 are mostly opposite, consistent with the regulator being in an inhibited state, while for 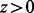 both signs are mostly the same, consistent with an activated regulator.

In the following, we take a detailed look at the underlying null model: Let 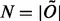 and the index 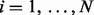 runs over all nodes 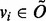. We consider the product 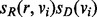 as being represented by independent and identically distributed random variables 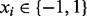 where both values −1 and 1 have equal probabilities 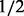. Expectation value and variance of *x_i_* are then given by 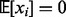 and 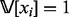. Let 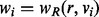 be the weights of the edges connecting *r* to *v_i_*. Setting now 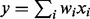 with 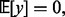 and 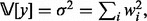 and noting that 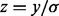 it is seen that approximately 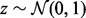 for large enough *N*.

The validity of *z*(*r*) to test for statistical significance of the regulator depends on whether the null model described above is deemed appropriate. Consider the extreme case where all dataset genes are up-regulated and the regulator *r* has only activating downstream connections. Assuming for simplicity that all weights are equal to 1, we then have 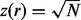 which is >2 for 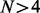. However, this would not be perceived as a significant match since any regulator with only activating downstream edges (and 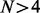) would come up as significant for this dataset. A better null model for calling significant regulators is based on randomizing the data rather than randomly flipping regulation directions. One possibility is to permute labels of genes in *D* while keeping their regulation direction and the size of the overlap *N* constant. This can be achieved approximately by skewing the distribution of the random variables *x_i_* defined above, i.e. by setting their expectation value *μ* to a non-zero value 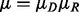 where 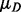 (or 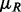) is given by the expectation value of the sign when randomly (and independently) picking a dataset gene (or an edge downstream of *r*). In particular we have 
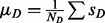
 and 
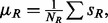
 where the sums run over all *N_D_* dataset genes, and over all *N_R_ r*-regulated genes. For the approximation to be valid, we also assume that 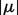 is not too close to 1, and for simplicity we keep the weights *w_i_* fixed.

The activation *Z*-score *z*(*r*) reflects a reasonable null model for calling significant regulators if the expectation value *μ* is sufficiently close to zero. In practice, we flag all regulators where 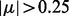 to indicate that the calculated *Z*-score should not be used for significance calls. In the case when the regulation directions of the dataset and downstream causal edges are skewed, it is possible to define a ‘bias-corrected’ *Z*-score (described in the Supplementary Material and available as an option in IPA) that can be used to determine statistically significant regulators.

### 3.4 Mechanistic Networks: inferring likely causal mechanisms

Regulators determined with URA are not necessarily independent of each other. It is for instance possible that the causal effect of a regulator *r*_1_ on the dataset is relayed through another regulator *r*_2_, with both regulators *r*_1_ and *r*_2_ coming up as independent significant hits. One indication for this is that *r*_1_ and *r*_2_ are themselves connected by a causal edge 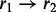 in *G*. The goal of the MN algorithm is to determine those network edges between pre-determined upstream regulators for which there is statistical evidence that the corresponding relationship is likely relevant for the causal mechanism behind the dataset. The most significant causal edges between regulators are then used to construct networks downstream of a ‘master’ regulator in order to indicate possible causal (e.g. signaling) mechanisms.

**Fig. 2.**
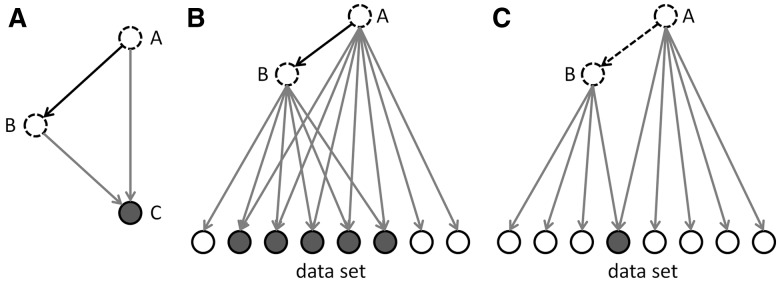
Enrichment of ‘causal transitive triangles’ (A) indicates causal dependency of upstream regulators A and B [compare (B) versus (C); see text]

The algorithm is based on the following idea: if the causal effect of *r*_1_ on some data set molecule 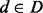 is transmitted through the intermediate regulator *r*_2_, we expect an elevated occurrence of cases where all three edges, 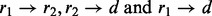 are present in the network, and the edge 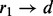 is explained by the path 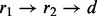. We therefore look for statistical enrichment of these ‘causal transitive triangles’ (Fig. 2A). This enrichment is given by the intersection of the overlaps of the regulated gene sets 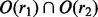 in the dataset (Fig. 2B and C), i.e. we compute the FET *P*-value with 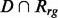 serving as the universe. These FET *P*-values are calculated for every edge 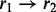 in *G* for which the regulators *r*_1_ and *r*_2_ meet pre-defined cut-offs with respect to their overlap *P*-value *p*(*r*) and activation *Z*-score *z*(*r*). For every upstream regulator *r*, the MN algorithm then constructs downstream networks with predefined ‘breadth’ *N* and ‘depth’ *K* from significant causal edges that connect *r* to dataset genes through several links by using the following recursive algorithm.

1. Starting from any upstream regulator *r*, select *N* regulators *s_i_* that are connected downstream through causal edges with lowest edge *P*-values.
2. For each *s_i_*: if maximal path length *K* is reached or a cycle is detected, skip, else set 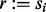 and go to (1).
3. Build network from union of all traversed edges.

Through the inference of likely relevant causal relationships the MN algorithm makes a prediction about which previously identified regulators are closer to the dataset genes than others in a causal chain of events. However, it is possible that the constructed networks are incomplete because not enough statistical evidence could be collected for missing edges. The algorithm does not enforce consistency of the predicted activation states and also accepts protein–protein binding edges as causal connections between upstream regulators.

### 3.5 Causal Network Analysis: exhaustive enumeration of possible root causes

The CNA algorithm generalizes URA by including paths from regulators to regulated molecules that involve more than one edge. For a given ‘root’ regulator *r* this is done by constructing shortest paths up to a certain length from *r* to dataset molecules, and constructing networks from the union of those paths in order to build mechanistic hypotheses. Hypothesis networks are then statistically scored using overlap *P*-value and activation *Z*-scores, and the activation state of the root regulator is determined. In contrast to URA, for simplicity, we are not taking continuous edge weights into account, but instead set all edge weights to 1 if they pass a predefined cut off *δ* (set to 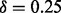 in the implementation). We only consider edges with non-ambiguous directions of regulation, i.e. non-zero sign *s*(*e*). The algorithm operates on the multigraph 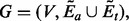 where the included sets of A- and T-edges are given by 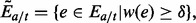 and the set of regulated genes (through T-edges) is 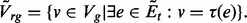


For each node 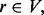 we construct all shortest paths *P* from *r* to every gene 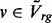 in *G*. These paths with length *K* are represented by sets of edges 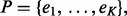 where 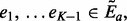 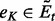 and 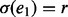, 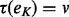, 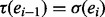. For any given path *P*, we define the sign

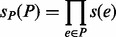

which represents the composite direction of regulation for a signal passed through the path as a causal sequence of events.

**Fig. 3.**
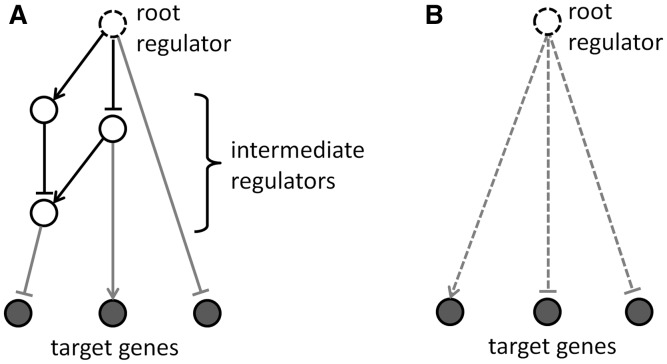
Replacing multi-step paths from root regulators to target genes (A) by ‘virtual’ edges with the same net effect (B). The pointed arrowheads represent activating (+1) relationships, and the blunt arrowheads represent inhibitory relationships (–1). The dashed lines indicate virtual relationships composed of the net effect of the paths between the root regulator and the target genes

For each pair of nodes 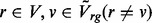 with at least one shortest path *P* connecting *r* and *v*, we define a ‘virtual’ edge 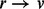 representing all shortest paths connecting *r* and *v* if all those paths are consistent, i.e. have the same net effect *s_P_* (Fig. 3A and B). We then collect all virtual edges corresponding to paths with length *K* in a set *E_K_*. Note that 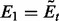 and all sets *E_K_* are disjoint. The sign 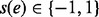 of 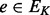 is then defined by 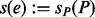 where *P* is any shortest path associated with *e*. We proceed by constructing graphs *G_K_* composed of virtual edges, representing paths with maximal length *K*:

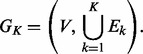

Note, that the *G_K_* are nested subgraphs of each other, i.e. 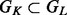 if 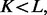, with the special case 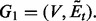

For every potential regulator 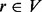 the algorithm computes overlap *P*-values 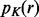 and activation *Z*-scores 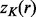 by applying URA (with all edge weights set to 1) independently to all networks *G_K_*. Hypothesis networks are then constructed from the union of all paths *P* connecting *r* to the dataset molecules. For any given regulator *r* there may be multiple hypotheses corresponding to the different values of *K*. These hypotheses again represent nested subgraphs with their sets of regulated genes given by 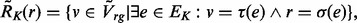 and 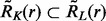 if 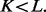 In practice we only construct hypotheses with 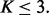


In order to limit the number of networks returned by the algorithm we only include hypotheses that add substantial information to the ‘sub’-hypotheses that are contained in the same network, i.e. in the spirit of Occam’s razor preference is given to simpler networks. In particular, we require that (i) the overlap *P*-value of a hypothesis is more significant than the *P*-value of a contained hypothesis, 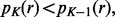 and (ii) the set of regulated molecules is different from the set of molecules targeted by any individual sub-network.

We note that all constructed hypothesis networks are completely consistent with respect to their direction of edge effects, and may contain loops only if a regulator node appears as a dataset molecule in *D* at the same time.

### 3.6 Network-bias-corrected *P*-value

Overlap *P*-values *p*(*r*) calculated using the method described in Section 3.3 may be skewed, i.e. may appear too significant because of the presence of network hub genes in the dataset. Hub genes are connected to many upstream regulators so their occurrence in a hypothesis network is less surprising. To correct for this network bias, we also calculate a network-bias-corrected *P*-value that measures significance of the overlap between the dataset and regulated genes by comparing to overlaps of random datasets with distributions of in-degrees similar to the actual dataset, and therefore preserving network topology. To perform this statistic, we divide the sets of regulated genes *V_rg_* or 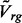 into bins containing only genes whose in-degree lies within a certain range. We sample independently from each bin such that the number of genes drawn corresponds to the number of dataset genes that fall in that bin. The (right-sided) *P*-value is then calculated from the distribution of the overlap with the hypothesis-regulated genes as before. If there is only one bin containing all genes, the resulting *P*-value is the regular FET overlap *P*-value defined in Section 3.3. If the number of bins is >1, this *P*-value needs to be calculated numerically by explicit permutation sampling. In our implementation, this is done using at most 10 000 independent permutations of genes (fewer if the estimated *P*-value > 0.01). The minimum *P*-value that can be computed this way is therefore 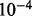. The computation of the network-bias-corrected *P*-value uses bins based on a logarithmic scale with base 2, so the ranges of in-degrees are {1}, {2,3}, {4, … ,7}, {8, … ,15}, {16, … ,31}, etc.

### 3.7 Downstream Effects Analysis

The goal of DEA is to identify those biological processes and functions that are likely to be causally affected by up- and down-regulated genes. In addition, it is also predicted whether those processes are increased or decreased. The approach is very similar to that of URA, except that the direction of edges connecting the dataset genes with the predicted entity (here, the biological process or disease) is reversed. The calculation of the overlap *P*-value is essentially the same as in standard enrichment functional analysis (an existing IPA feature).

For the calculation of the activation *Z*-score, we consider the graph 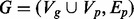 as the underlying network, and define edge signs and weights as 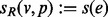 and 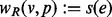 where 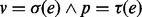 and 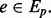 For any given process *p*, the set of genes regulating that process is

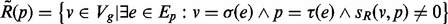

and the overlap with the dataset is given by

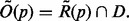

The activation *Z*-score *z*(*p*) is then given by the corresponding formula in Section 3.3 and its sign is used to predict whether the downstream process is increased or decreased.

## 4 IMPLEMENTATION

IPA enables end-users to execute URA, MN, DEA and CNA prediction algorithms when analyzing their datasets. In this web application, the user selects a gene-expression dataset to analyze, and specifies several optional settings that will tailor the analysis as desired. After the analysis job is submitted, IPA performs a number of different calculations on the dataset, including the prediction algorithms, and produces an analysis result. This result can be viewed within IPA and it displays conclusions in a variety of ways, depending on the algorithm.

**Fig. 4.**
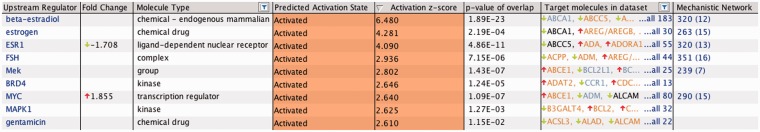
URA result table for example (1) in Section 5.1 (beta-estradiol-treated MCF-7 cells)

URA is always executed as part of IPA’s dataset analysis, and there are no options to choose before running the analysis. The results are displayed in a table in which each row shows information about a particular regulator and the molecules in the dataset that the regulator targets (see example in Fig. 4). Columns for predicted state, *Z*-score, and *P*-value enable users to identify regulators of interest. The table can be sorted and filtered, and toolbar operations support the creation of network diagrams and lists based on items in the table.

**Fig. 5.**
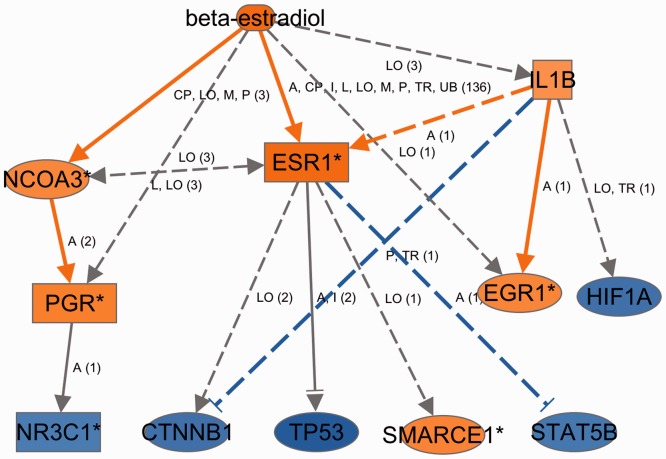
Mechanistic network for beta-estradiol [example (1) in Section 5.1]. In this network, beta-estradiol is postulated to activate ESR1 (the estrogen receptor), NCOA3 (a key estrogen receptor co-regulator) and to affect a number of other regulators to explain the gene-expression changes in the dataset. The set of regulators in total connect to 320 dataset genes (not shown), with beta-estradiol connecting directly to 183 of them

The MN algorithm is run upon the regulators from the URA results, and generated networks are accessible from the ‘Mechanistic Network’ column to the far right in the upstream regulator table. The column displays the number of dataset molecules targeted by the network followed in parenthesis by the number of regulators in the network. Clicking on the link in this column will display the corresponding network (see example in Fig. 5).

Unlike URA, the MN algorithm can be influenced by a variety of parameters. When the analysis is initially run, default values are used for these settings. After the initial run of the analysis, the user can re-run the algorithm with different values for *P*-value and *Z*-score cut offs, included relationship types, and parameters that control the shapes of the resulting networks. When the algorithm re-executes, the new mechanistic networks will replace the old ones in the result table.

Like the other prediction features, the CNA executes as part of a dataset analysis in IPA. Because of the large number of hypothesis networks that are typically returned, the CNA tool provides a means to automatically annotate each hypothesis for its connection to a particular biological concept such as a disease, phenotype, biological process, compound or gene. If the user enters such a concept into the tool, then after the hypotheses are created, IPA evaluates whether there are known relationships in the Ingenuity Knowledge Base between the root regulator of each hypothesis and that concept.

**Fig. 6.**
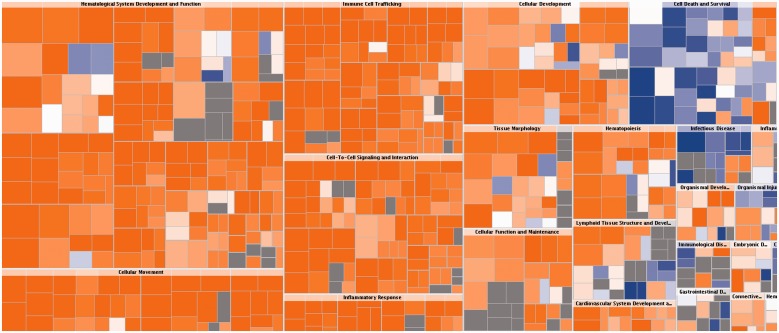
DEA results for example (2) in Section 5.2 (TNF-stimulated HUVEC cells). The visualization is a TreeMap (hierarchical heatmap) where the major boxes represent a family (or category) of related functions. Each individual colored rectangle is a particular biological function or disease and the color orange indicates its predicted state: increasing (orange), or decreasing (blue). Darker colors indicate higher absolute *Z*-scores. In this default view, the size of the rectangles is correlated with increasing overlap significance (using FET *P*-value). The image has been cropped for better readability

The results of DEA are displayed in a tree map (Shneiderman *et al.*,1992) (see Fig. 6 as an example) which clusters related functions together, thus providing a high-level view of the function families. The organization makes it easier to see cases where related functions are predicted to increase/decrease most significantly as a group. The user can then drill down to specific functions to see the predictions at a more granular level.

## 5 BIOLOGICAL RESULTS FOR EXAMPLE USE CASES

### 5.1 Upstream Regulator Analysis and Mechanistic Networks

One means to validate the effectiveness of the URA is to test its predictive capabilities using datasets derived from cells treated with a defined upstream stimulus. To this end, we retrieved from GEO (http://www.ncbi.nlm.nih.gov/geo) relevant gene-expression datasets which had not been curated by Ingenuity:

1. MCF-7 human breast cancer cells exposed to beta-estradiol, a well-known agonist of the alpha and beta estrogen receptors (the transcription factors ESR1 and ESR2 in humans). Retrieved from GSE11352 (Lin *et al.*, 2007).
2. Primary human endothelial cells (HUVEC) stimulated with the cytokine TNF. Retrieved from GSE2639 (Viemann *et al.*, 2006).

The raw microarray data files were processed through an automated feature extraction and normalization pipeline developed at Ingenuity Systems (and based on R/Bioconductor), and significantly differentially expressed genes were uploaded and analyzed in IPA. The three top-most predicted activated upstream regulators by *Z*-score for the dataset involving beta-estradiol treated cells are beta-estradiol itself, the more general parent compound estrogen, and the estrogen receptor (ESR1). A mechanistic network generated for beta-estradiol in this analysis (see Fig. 5) shows that it may exert its effects on the observed gene expression by interacting with ESR1 (as expected) and also through co-activators and other regulatory molecules. The set of 11 regulators plus beta-estradiol in total connects to 320 dataset genes.

The three top-most predicted inhibited upstream regulators (by negative *Z*-score, data not shown) are tretinoin, fulvestrant and 3-deazaneplanocin. Compounds like these that are predicted to be ‘inhibited’ produce expression patterns in the dataset partially or completely opposite to the effect observed in the literature. This can imply therapeutic utility for these compounds, by potentially reversing a phenotype observed in the disease state. Indeed, fulvestrant is a known selective estrogen receptor down-regulator approved for treatment of hormone receptor positive metastatic breast cancer. Tretinoin is the active metabolite of vitamin A (also known as all-trans retinoic acid) and is an RXR agonist that has been shown to inhibit the proliferation of MCF-7 cells and is indicated for certain cancers (Arteaga *et al.*, 2013; Wang *et al.*, 2000; Zhu *et al.*, 1997). It may affect proliferation in MCF-7 cells by interfering with the estrogen receptor’s ability to activate its downstream target genes (Ombra *et al.*, 2013) or through other means (Muller *et al.*, 2002). 3-deazaneplanocin A (also known as DZNep) affects histone methylation and recent work suggests it might have therapeutic value for breast cancer (Hayden *et al.*, 2011).

In the second example of the HUVEC cells treated with the cytokine TNF, the top-predicted activated regulators are TNF itself, followed by lipopolysaccharide, NF-κB complex, IFNG and poly rI:rC-RNA. Several of these regulators initiate an inflammatory response much like TNF does (i.e. lipopolysaccharide and poly rI:rC-RNA), and NF-κB is the key downstream transcription factor activated when TNF activates its receptor. To further validate the algorithm, we retrieved nine additional gene-expression datasets from GEO all derived from mammalian cells directly exposed to TNF. The 10 datasets in total come from human, mouse, rat and cow, and include a variety of cell types such as fibroblasts, epithelial cells and monocytes, and are from several technology platforms including Affymetrix, Agilent and Illumina (RNA-Seq). In eight of 10 of these datasets, IPA predicted TNF as the top-activated regulator, and ranked it second and third in the other two datasets (see Supplementary Tables). This high level of consistency lends support to the premise that the URA has predictive power across mammalian cell types and species, and is independent of expression measurement technology.

### 5.2 Downstream Effects Analysis

To highlight the utility of DEA we examined its predictions for the HUVEC cells treated with TNF. As shown in Figure 6, a large number or biological processes are predicted to be increased by TNF, especially those in the ‘Hematological System Development and Function’, ‘Cellular Movement’, ‘Immune cell Trafficking’, ‘Cell-to-Cell Signaling and Interaction’ and ‘Inflammatory Response’ categories. Examples of specific increased function among those categories are ‘leukocyte migration’ (*Z*-score 5.006), ‘inflammatory response’ (*Z*-score 4.578) and ‘quantity of T lymphocytes’ (*Z*-score 3.934). Increases in these types of biological functions are expected for TNF.

### 5.3 Causal Network Analysis

The power of CNA is its ability to detect novel master upstream regulators that operate though other regulators, especially in cases where few or no relationships exist directly between it and the dataset genes. To highlight this capability we obtained a gene-expression dataset derived from a mouse model of ankylosing spondylitis (Haynes *et al.*, 2012). This disease is characterized by initial inflammation that progresses to osteoproliferation leading to inappropriate bone formation and eventually joint fusion. The pathology can be produced in mouse by injecting human cartilage proteoglycan extract several times over a 12-week period by which time inflammation leads to bone overgrowth in the spine.

**Fig. 7.**
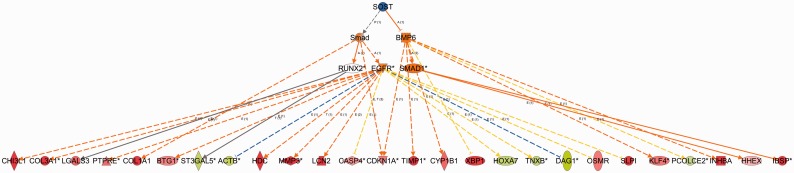
CNA result for SOST (see Section 5.3). SOST is the master regulator (or ‘root’ regulator) of a small causal network containing five intermediate regulators that may explain the up and down regulation of the 26 dataset molecules shown in the bottom row (red indicates up-regulation and green down-regulation). The regulators are colored by their predicted activation state: activated (orange) or inhibited (blue). Darker colors indicate higher absolute *Z*-scores. The edges connecting the nodes are colored orange when leading to activation of the downstream node, blue when leading to its inhibition, and yellow if the findings underlying the relationship are inconsistent with the state of the downstream node. Pointed arrowheads indicate that the downstream node is expected to be activated if the upstream node connected to it is activated, while blunt arrowheads indicate that the downstream node is expected to be inhibited if the upstream node that connects to it is activated

The authors sought to find evidence in the expression data that the Wnt signaling system was perturbed. By using RT-PCR and immunohistochemistry they were able to show that the Wnt pathway inhibitory molecules DKK1 and SOST were slightly reduced in concentration both at the mRNA and protein levels in the spinal tissue. Using CNA in IPA with the authors’ microarray-based mRNA expression data as input, we found that SOST was predicted to be significantly inhibited with a *Z*-score of −1.96, with a network depth of 3, meaning that some paths from SOST to the dataset molecules involve three distinct ‘hops’ (with two intervening regulators), such as the path SOST 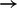 Smad 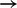 EGFR 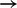 LCN2 as shown in Figure 7. The gene for SOST itself was not detectably differentially expressed in this experiment based on the microarray data, highlighting the sensitivity of the causal analysis.

As a point of interest, the canonical pathway in IPA which shares the highest overlap with the regulators in this causal network is the pathway called ‘Role of Osteoblasts, Osteoclasts and Chondrocytes in Rheumatoid Arthritis’, (containing SOST, the Smad family, SMAD1, BMP6, and RUNX2), indicating the relevance of SOST and its downstream targets both in bone homeostasis and inflammatory disease.

## 6 DISCUSSION

This article describes algorithms, tools and visualizations recently added to IPA that enable scientists to combine the directional information encoded in their gene-expression datasets with knowledge extracted from the literature to infer the underlying causes of their observed transcriptional changes and to predict likely outcomes. This is a significant advance compared to tools that just look for statistical enrichment in overlap to sets of genes. An aspect of upstream analysis that makes it especially powerful is that it is essentially an activity-based prediction, in that the measurement of the activity of a transcriptional regulator is via the measurement of genes known to be differentially expressed by it in a defined direction. The data being analyzed has measured that differential expression. This can be contrasted with for example pathway overlap prediction, where in general there is no guarantee that the members of the pathway are differentially expressed upon pathway activation or inhibition.

As described above, the algorithms operate over a large-scale causal graph assembled from individual literature-supported relationships between molecules, diseases and biological processes in the Ingenuity Knowledge Base. These relationships are derived from a myriad of experimental systems in mouse, rat and human. Though possibly counter-intuitive to mix relationships derived from a variety of contexts to analyze data from a particular context, the results are generally valid due to the conserved activity of most genes and proteins across tissues and cell types. That being said, it may be possible in the future to automatically infer a context based on the expression data and to filter accordingly to only relevant relationships for a particular analysis.

There are at least 30 papers that have already made use of these new causal tools in IPA. One of the first to use URA demonstrated how transcription factor activation differs between mouse, macaque, and swine in response to infection by the 2009 pandemic H1N1 influenza virus (Go *et al.*, 2012). Another used both URA and DEA to help characterize the mechanisms that provide breast cancer protection during pregnancy (Meier-Abt *et al.*,2013). There are certain to be additional applications of these powerful analytics as scientists discover and adopt them in their research.

## Supplementary Material

Supplementary Data
