# Supplementary material for: Causal analysis approaches in Ingenuity Pathway Analysis
Source: Bioinformatics. 2013 Dec 13;30(4):523–30. doi: 10.1093/bioinformatics/btt703 (PMC3928520; doi:10.1093/bioinformatics/btt703)
Supplement: Supplementary Data [file supp_30_4_523__index.html]

Causal analysis approaches in Ingenuity Pathway Analysis — Causal analysis approaches in Ingenuity Pathway Analysis — Supplementary Data 

# Causal analysis approaches in Ingenuity Pathway Analysis

## Supplementary Data

files

**Files in this Data Supplement:**

- Supplementary Data - pdf file
- Supplementary Data - xlsx file
