# Supplementary material for: Causal analysis approaches in Ingenuity Pathway Analysis
Source: Bioinformatics. 2013 Dec 13;30(4):523–30. doi: 10.1093/bioinformatics/btt703 (PMC3928520; doi:10.1093/bioinformatics/btt703)
Supplement: Supplementary Data [file supp_btt703_supplementary_material.pdf]

Causal Analysis Approaches in Ingenuity Pathway Analysis (IPA) (Supplementary Material)

Andreas Krämer, Jeff Green, Jack Pollard, Jr., Stuart Tugendreich

Finding and edge types in the causal network (table)

The following table shows the association of the various finding categories in the Ingenuity Knowledge Base with one of the three edge types (T, A, or P) present in the causal network. The edge signs shown are associated with an increase of the corresponding process. Signs are reversed if the process is decreased.

findings category/process	edge type	sign
expression (E)	T	1
transcription (T)	T	1
protein-DNA binding (PD)	T	0
activation (A)	A	1
inhibition (I)	A	-1
phosphorylation (P)	A	1 or -1
ubiquitination (Ub)	A	-1
molecular modification (M)	A	0
causal molecule/function relationship (C)	P	1

Edge weights and signs

The assignment of regulation directions to edges is in general not unambiguous because the underlying findings can be inconsistent. This is not surprising since findings represent independent experimental observations that were obtained in different experimental contexts (e.g. organism, cell line, or more complex situations). Ideally one would only consider findings that are applicable to the biological context of the gene expression data under consideration, but this would often make the network too sparse to allow for meaningful causal analysis. We take the approach of using weights assigned to edges that reflect our confidence in the assigned direction of the causal effect. Less weight is put on edges with fewer findings or ambiguous direction of regulation. The idea is that a higher weight (i.e. the presence of many consistent findings in diverse contexts) makes it more likely that the given direction of regulation is also applicable to the context at hand. In particular we define the sign $s(e) \in \{-1, 0, 1\}$ and weight $w(e) \in [0, 1)$ of an edge $e \in E$ as

$$s(e) = \operatorname{sgn}\left(\sum_{f \in F(e)} \tilde{s}(f)\right)$$

$$w(e) = \frac{1}{N+1}\left|\sum_{f \in F(e)} \tilde{s}(f)\right|$$

Bias-corrected z-score

We can define a “bias-corrected” z-score by

$$z_{\text{bias-corrected}}(r) = \frac{\sum_{v \in \tilde{O}} w_R(r,v)\,[s_R(r,v)s_D(v) - \mu]}{\left(\sum_{v \in \tilde{O}} [w_R(r,v)]^2\right)^{1/2}}.$$

This is seen by setting $y = \sum_i w_i x_i$, leading to $\mathbb{E}[y] = \mu \sum_i w_i$, and $\mathbb{V}[y] = \sigma^2 = (1-\mu^2)\sum_i w_i^2 \approx \sum_i w_i^2$ if $\mu^2 \ll 1$. It follows that $z = (y - \mu \sum_i w_i)/\sigma \sim \mathcal{N}(0,1)$ for sufficiently large N.

Description of Ingenuity Pathway Analysis (IPA)

IPA® is a web-based software application that enables researchers to analyze data derived from expression and SNP microarrays, RNA-sequencing, proteomics and metabolomics experiments, and small-scale experiments (such as PCR) that generate gene or protein lists. It also allows search for targeted information on genes, proteins, chemicals, diseases, and drugs, as well as building your own biological models. IPA's data analysis and experimental modeling enables understanding the significance of data or target(s) of interest in relation to larger biological or chemical systems. Importantly, IPA's causal analytics are made possible by the Ingenuity® Knowledge Base, a uniquely structured repository of biological and chemical "findings" curated from various sources including the literature. Literature findings are individually and manually curated and modeled ontologically by trained Ph.D.-level curators, and include the direction of the effect from the upstream to downstream molecule in a molecular relationship when it is stated in the paper. For example, the following finding from the Ingenuity Knowledge Base describes how the TNF protein increases expression of the TSG6 gene:

> *"In smooth muscle cells from human cervix, TNF-alpha [TNF] protein increases (in a time-dependent and dose-dependent manner) expression of human TSG6 [TNFAIP6] mRNA"*

curated from

> Fujimoto T, Savani RC, Watari M, Day AJ, Strauss JF. Induction of the hyaluronic acid-binding protein, tumor necrosis factor-stimulated gene-6, in cervical smooth muscle cells by tumor necrosis factor-alpha and prostaglandin E(2). *Am J Pathol* 2002 Apr 1;i **160**(4):1495-502. (PubMed ID 11943733)

The finding includes contextual details such as the species, cell and tissue type, and the fact that it was found to be time and dose dependent. The Ingenuity Knowledge Base currently contains approximately 5 million individual findings, most of which describe relationships between molecules or between molecules and diseases or biological functions. Freely-available software to perform gene expression analysis such as DAVID (http://david.abcc.ncifcrf.gov/tools.jsp), GeneMania (http://www.genemania.org), CytoScape (http://www.cytoscape.org) and others are useful, but lack the detailed and directional molecular information available to IPA. Therefore, while these tools can compute statistical associations to biological concepts (such as to biological functions as categorized by GO (http://www.geneontology.org)), in our opinion, they cannot support IPA's causal inferencing methods that are described in the present paper. IPA is developed, maintained and updated continuously. Additional information can be found on Ingenuity Systems' website (http://www.ingenuity.com).
